# Angiotropism and extravascular migratory metastasis in cutaneous and uveal melanoma progression in a zebrafish model

**DOI:** 10.1038/s41598-018-28515-6

**Published:** 2018-07-11

**Authors:** Giulia Fornabaio, Raymond L. Barnhill, Claire Lugassy, Laurent A. Bentolila, Nathalie Cassoux, Sergio Roman-Roman, Samar Alsafadi, Filippo Del Bene

**Affiliations:** 1 Institut Curie, PSL Research University, Inserm U934, CNRS UMR315, F-75005 Paris, France; 2 Institut Curie, PSL Research University, Department of Translational Research, F-75005 Paris, France; 3 Sorbonne Universités, UPMC University Paris 6, CNRS UMR 3215, INSERM U934, F-75005 Paris, France; 4 Institut Curie, PSL Research University, Department of Pathology, F-75005 Paris, France; 5 Faculty of Medicine, University of Paris René Descartes, F-75006 Paris, France; 6 California NanoSystems Institute, Los Angeles, CA 90095 USA; 7 Department of Chemistry and Biochemistry, University of California, Los Angeles, CA 90095 USA; 8 Institut Curie, PSL Research University, Department of Ophthalmology, F-75005 Paris, France

## Abstract

Cutaneous melanoma is a highly aggressive cancer with a propensity for distant metastasis to various organs. In contrast, melanoma arising in pigmented uveal layers of the eye metastasizes mostly in the liver. The mechanisms of these metastases, which are ultimately resistant to therapy, are still unclear. Metastasis via intravascular dissemination of tumour cells is widely accepted as a central paradigm. However, we have previously described an alternative mode of tumour dissemination, extravascular migratory metastasis, based on clinical and experimental data. This mechanism is characterised by the interaction of cancer cells with the abluminal vascular surface, which defines angiotropism. Here, we employed our 3D co-culture approach to monitor cutaneous and uveal human melanoma cells dynamics in presence of vascular tubules. Using time-lapse microscopy, we evaluated angiotropism, the migration of tumour cells along vascular tubules and the morphological changes occurring during these processes. Cutaneous and uveal melanoma cells were injected in zebrafish embryos in order to develop xenografts. Employing *in vivo* imaging coupled with 3D reconstruction, we monitored the interactions between cancer cells and the external surface of zebrafish vessels. Overall, our results indicate that cutaneous and uveal melanoma cells spread similarly along the abluminal vascular surfaces, *in vitro* and *in vivo*.

## Introduction

Metastasis is described as the spread of cancer cells from the original (primary) tumour to a different (secondary) site. Metastasis via intravascular or intralymphatic dissemination of tumour cells is widely accepted as a central paradigm. However, in our previous work, we have described an alternative mode of tumour dissemination without intravasation, namely extravascular migratory metastasis (EVMM), based on clinical^1,2^ and experimental^3,4^ data in cutaneous melanoma and on clinical data in uveal melanoma^5^. This mechanism is characterised by the interaction of tumour cells with the external vascular surface, which defines angiotropism. This process is described histologically by the presence of tumour cells disposed along the external surface of vascular structures in a ‘pericytic location’ without intravasation^1^. Since the very first description of angiotropism, it was stressed that angiotropic melanoma cells were connected to the endothelium thanks to an amorphous basement membrane containing laminin^6^. Interestingly, it is possible to identify angiotropism either at the invasive front of the tumoural mass or in neighbour tissues. Angiotropism promotes pericytic mimicry, the replacement of pericytes by tumour cells spreading along the abluminal surfaces of vessels, as exhibited in various *in vitro* and *in vivo* models^3,4,6^. Employing our 3D co-culture model of pericytic mimicry and angiotropism^7^, we have previously demonstrated that the interaction between endothelial cells and cutaneous melanoma cells triggered differentially-expressed genes linked to cancer progression; interestingly, ten of these genes were also associated with inflammation^6^. Notably, in a collaborative study we showed that UV-induced inflammation promotes pericytic mimicry, angiotropism and eventually metastasis in a genetically engineered murine melanoma model^4^. The current study corroborates the involvement of processes such as angiotropism and pericytic mimicry in cutaneous and uveal melanoma progression and metastasis.

Both cutaneous and uveal melanomas are derived from melanocytes, which originate from the neural crest. Despite this common origin, cutaneous and uveal melanomas show two distinct genetic profiles. Cutaneous melanoma is regarded as one of the most serious forms of skin cancer because it may metastasize to many distal organs, such as the lungs, liver and brain. Originally, it was considered as a homogeneous condition with a generally poor prognosis, but further and more detailed studies led to the description of a number of distinct subtypes with diverse clinicopathological peculiarities. In particular, four principal subtypes were identified, based on the preferred site of origin of the tumour, relative amount of ultraviolet (UV) light exposure and duration of pre-invasive growth. These are superficial spreading, nodular, lentigo maligna, and acral lentiginous melanomas^8^. BRAF and NRAS genes, which encode mitogen-activated protein kinase (MAPK) pathway constituents, are recurrently mutated in cutaneous melanoma. The frequency of BRAF and NRAS mutations differs among the cutaneous melanoma subtypes^9^.

Uveal melanoma is the most common intraocular malignancy and arises in the pigmented layers of the eye. Up to 50% of the patients develop metastasis, mostly in the liver (approximately 90%). Primary uveal melanoma can be cured by surgery (enucleation) or radiotherapy, but the metastatic setting is refractory to treatments^10^. Compared to other solid tumours such as cutaneous melanoma, uveal melanoma shows a remarkably low mutation burden; indeed, it does not display the UV radiation DNA-damage signature, observed in the majority of melanoma. Mutually exclusive mutations in GNAQ or in GNA11, the principal driver oncogenes in uveal melanoma, occur in approximately 85% of cases^11–13^. Moreover, inactivating mutations in the tumour suppressor BAP1 occur in ~85% of metastatic tumours and are associated with disease dissemination^14^ and poor prognosis.

In this study, employing our 3D co-culture approach^7^ and time-lapse microscopy, we evaluated angiotropism and migration of cutaneous and uveal melanoma cells along the vascular tubules and the morphological changes occurring during these processes.

Cutaneous or uveal melanoma cell lines were injected in 2-days-post-fertilization (dpf) zebrafish embryos in order to develop xenograft models of human cutaneous and uveal melanoma.

The zebrafish could be considered as an excellent model system for our purpose, due to various useful characteristics^15^. Specifically focusing on cancer, zebrafish has the benefit to show high conservation of tumour suppressor genes and (proto-) oncogenes with humans; for this reason, it is an ideal model to identify both novel therapeutic compounds and clinically relevant genes^16,17^. Moreover, zebrafish xenografts show histopathological and gene-profiling features similar to the ones of human tumours^18^. In addition, the adaptive immune system of zebrafish reaches maturity just after 4 weeks post fertilization^19,20^; therefore, the use of zebrafish at embryonic and larval stages permits to avoid graft rejections. This feature has been previously exploited to perform xenotransplantation with human cancer cells, including melanoma cells, without immunosuppression^21–23^. Moreover, the availability of different tissue-specific fluorescent reporter transgenic lines, combined to the optical transparency of zebrafish, permits the detection of tumour masses within one week and offers the possibility to perform high resolution, non-invasive live imaging of fluorescently labelled cancer cells^24–26^.

Employing *in vivo* imaging coupled with 3D reconstruction, we explored and monitored the interactions between cutaneous/uveal melanoma cells and the external surface of zebrafish vessels. Our work provides the first models of angiotropism and extravascular migratory metastasis of cutaneous and uveal melanoma in zebrafish.

## Methods

### Animal care and handling

In this study, we used the zebrafish transgenic line *Tg*(*kdrl:Hsa*.*HRAS-mCherry*), expressing mCherry in endothelial cells^27^. The animals were maintained according to standard protocols (http://ZFIN.org). All the experiments were performed in accordance to the European and the French National Regulation for the Protection of Vertebrate Animals used for Experimental and other Scientific Purposes (Directive 2010/63; French Decree 2013–118). According to this Directive, early life-stages of zebrafish are not protected as animals until the stage of being capable of independent feeding, namely after 5 days post fertilization, which is later than the endpoint of our experiments. This study was approved by the French Ministry of Higher Education and Research (Reference: APAFIS#6031-20 16070822342309 v2).

### Cell culture

The cutaneous melanoma cell line C8161-GFP^28^ was a gift from Dr. D. Welch (University of Kansas, Medical Center, USA). The uveal melanoma cell lines OMM 2.3 and OMM 2.5 were kindly provided by Dr. P.A. Van Der Velden (Leiden University, The Netherlands). They were both established in the Schepens Eye Research Institute (The Massachusetts Eye and Ear Infirmary, Harvard Medical School, Boston) from a liver metastasis in the same patient^29^. C8161-GFP cells were cultured in Dulbecco’s modified Eagle’s medium (Gibco, 41965-039) with 10% fetal calf serum and 1% Pen-Strep (Gibco, 15140-122) at 37 °C and 5% CO_2_. Human umbilical vein endothelial cells (HUVEC, Lonza C2519A) were grown in EBM-2 Endothelial Growth Basal medium (Lonza, CC-3156), supplemented with EGM-2 SingleQuots Kit (Lonza, CC-4176), at 37 °C and 5% CO_2_. OMM 2.3 and OMM 2.5 were cultured in RPMI-1640 medium (Gibco, 21875-034), with 10% fetal calf serum and 1% Pen-Strep (Gibco, 15140-122), at 37 °C and 5% CO_2_.

The non-malignant human melanocyte cell line Hermes 2B^30^ was kindly provided by Prof. D. Bennett (St George’s, University of London, UK). Hermes 2B were cultured in RPMI-1640 medium (Gibco, 21875-034), supplemented with 10% fetal calf serum, 200 nm 12-O-Tetradecanoylphorbol 13-acetate (Sigma, P8139), 200 pM Cholera toxin (Sigma, C8052) and 10 nM Endothelin 1 (Bachem, 6995), at 37 °C and 5% CO_2_.

OMM 2.3, OMM 2.5 and Hermes 2B cells were infected with lentiviral particles expressing the Green Fluorescent Protein (GFP) and a Puromycin-resistance cassette. The particles were obtained via triple transfection of HEK-293T cells with lentiviral plasmid pLVX-EF1α-AcGFP1-N1 (Clontech, 631983), packaging plasmid psPAX2 (Addgene, 12260) and VSV-G envelope expressing plasmid pMD2.G (Addgene, 12259). After the infection, the cells were selected for 3 days in complete growth medium containing 1.5 μg/mL Puromycin for OMM 2.3, 1 μg/mL Puromycin for OMM 2.5 and 10 μg/mL Puromycin for Hermes 2B. Infection of cells with GFP-Puromycin viruses did not noticeably mutate the morphology and the growth properties of the cells. All our experiments in zebrafish were performed with respect to the restrictions of use of these cell lines.

### 3D co-culture matrigel assay

For the endothelial tubule model assay, 24 wells plates (TPP Z707791) were coated with 100 μl of basement membrane extract (Cultrex PathClear, Trevigen 3533-001-02) and incubated at 37 °C for 30 min to promote the polymerization of the gel. 40,000 human endothelial cells (HUVEC, Lonza C2519A) were added to each well and let form the tubular structures overnight. After 12-14 hours, 10,000 C8161-GFP, OMM 2.3-GFP, OMM 2.5-GFP or Hermes 2B-GFP cells were carefully plated on the endothelial tubules network. The co-cultures were then incubated approximately 2 hours at 37 °C and subsequently imaged up to 24 hours, using IncuCyte® S3 Live-Cell (ESSEN Bioscence), employing a dry 20 × objective. Images were then processed with the Incucyte, ImageJ and Adobe Illustratore software.

### Human cancer cells injections in zebrafish embryos

Before injections, zebrafish embryos were kept at 28 °C and manually dechorionated few hours before the injection. Melanoma cells were grown to 80–90% confluency, washed one time with PBS and trypsinised (0.25% trypsin/0.53 mM EDTA) to obtain a single cell suspension. Cells were then centrifuged for 5 minutes at 1000 g and resuspended in RPMI-1640 (for uveal melanoma lines and Hermes 2B) or DMEM medium (for cutaneous melanoma lines), in order to reach a final concentration of 6,000–10,000 cells/μl. At 2 dpf, the larvae were anesthetised with 0.004% tricaine and positioned flanking on one side on a 10-cm Petri dish coated with 2% agarose. Cells were injected using glass capillary needles with an approximate opening equal to the dimension of one cell. 300 to 500 cells were injected into the yolk sac, using a pneumatic pico pump and a manipulator. The number of cells was determined by measuring the size of the drop of suspension injected. After the injection, embryos were incubated to recover for at least one hour at 28 °C and then maintained in egg fish water at 34 °C. This temperature was chosen as an intermediate temperature between 37 °C (optimal for cell lines) and 28 °C (optimal for zebrafish embryos and larvae), in order to permit a normal development of the larvae^21^, without impairing melanoma growth and migration.

### Microscopy

Starting from 30 hours post injection (hpi), embryos showing melanoma cells that had migrated were selected using a Leica MZ FLIII stereomicroscope (Leica) equipped with a Leica DFC310FX digital camera (Leica). Zeiss LSM 700 confocal microscope or Zeiss LSM 880 microscope (Zeiss) were used, employing a 25 × oil, a 40 × water immersion or a 63 × water immersion objective. For confocal microscopy, larvae were anesthetised in egg fish water containing 0.02% tricaine, immobilised in 1.2% low-melting agarose and then imaged up to 12 hours (one acquisition every 15–25 minutes), using 488 and 568 nm lasers. *Z*-volumes were acquired with a 1- to 2-μm resolution and images were processed using ImageJ, Imaris-Bitplane, Zen Blue and Adobe Illustrator software.

### Histopathology of human cutaneous and uveal melanoma samples

Formalin-fixed-paraffin-embedded (FFPE) 5-μm sections from primary cutaneous invasive melanoma and recurrent primary uveal melanoma were de-paraffinized and stained respectively with hematoxylyn eosin or with hematoxylin eosin saffron.

The cutaneous melanoma lesion was excised from the upper back of a 33-year-old woman. The histopathologic examination of this lesion revealed a primary cutaneous invasive melanoma of the superficial spreading type, with Breslow thickness of 0.84 mm, absence of ulceration and mitotic rate of 2 per mm^2^. By contrast, the uveal melanoma sample originated from the left eye of a 56-year-old woman, presenting a lesion of 16 mm in greatest diameter. Six years after radiation therapy the patient underwent enucleation for recurrent melanoma.

We confirm that all methods were performed in accordance with the relevant national guidelines and regulations. In accordance to the national law on the protection of individuals taking part in biomedical research, patients were informed by their referring oncologist that their biological samples could be used for research purposes and they gave their verbal informed consent. This study was approved by the institutional review board and ethics committee of the Institut Curie Hospital Group.

### Data availability statement

The datasets generated during and/or analysed during the current study are available from the corresponding author upon request.

## Results

### Angiotropism and extravascular migratory metastasis in cutaneous melanoma

**Figure 1:**
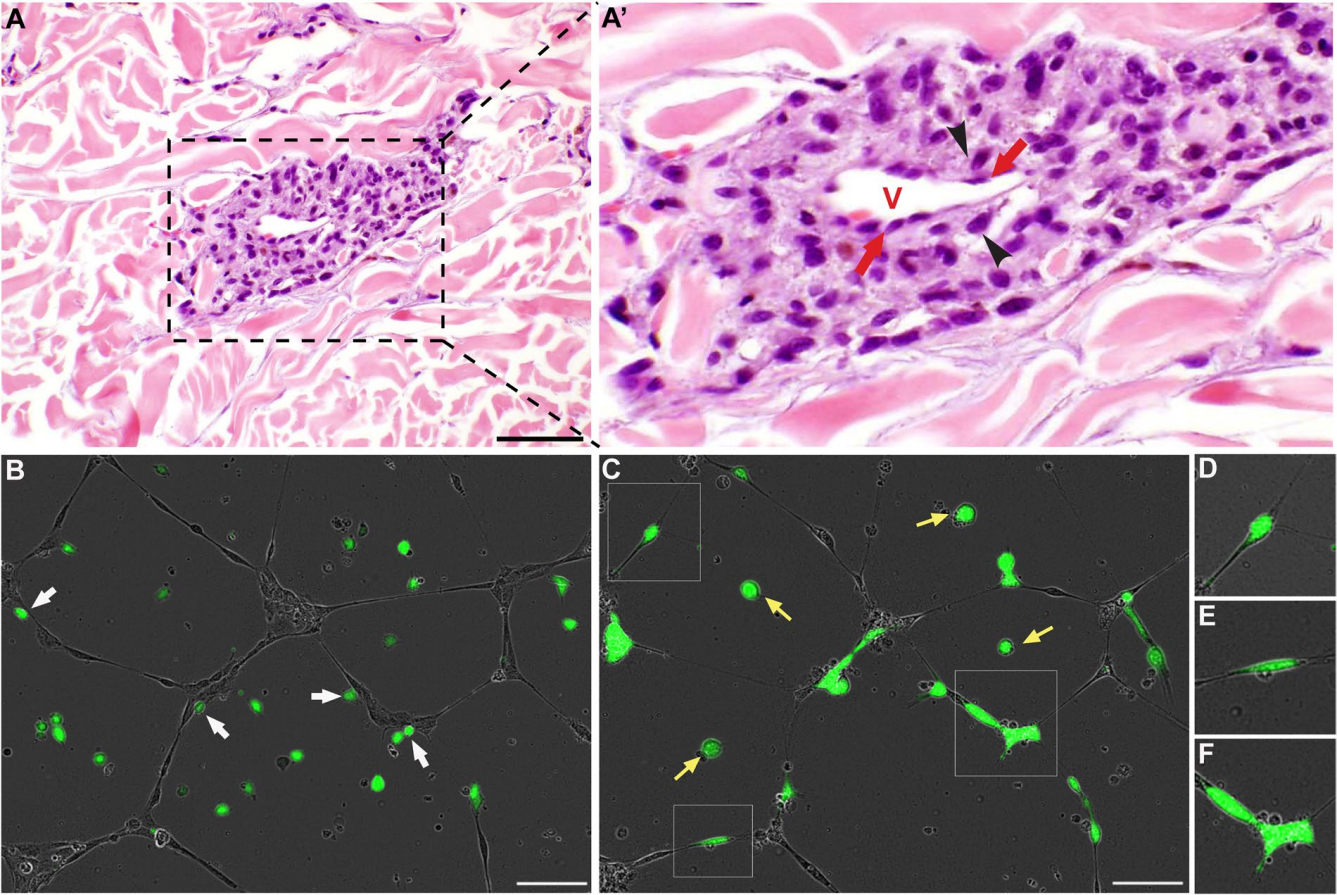
Angiotropism and extravascular migratory metastasis in cutaneous melanoma. (**A**) and (A’) Angiotropism in primary cutaneous invasive melanoma. In this field, a perivascular aggregate of angiotropic melanoma cells (black arrowheads) is disposed along the external endothelial surface of a microvessel. No melanoma cells are present within the vascular lumen, i.e., intravasation is absent. Black line = 50 µm, red V = lumen of the vessel, black arrowheads = melanoma cells, red arrows = endothelial cells. (**B**–**F**) C8161-GFP Cutaneous melanoma cells co-cultured with HUVEC Endothelial cells on BME. (**B**) Picture taken 2 h after the plating of C8161-GFP cells. (**C**) Picture taken 24 h after the plating of C8161-GFP, demonstrating the spreading of the melanoma cells along the endothelial tubules. (**D**–**F**) are zoomed-in images of angiotropic cells showed in image (**C**), in squares. (**B–F**) Time-lapse images of a 24-hour video. Scale bar is 100 µm, green cells are melanoma cells, grey cells are endothelial cells, white arrows show melanoma cells already attached to tubules at 2 h, yellow arrows show melanoma cells that did not attach to tubules, remaining rounded in the BME.

A clinical example of primary cutaneous invasive melanoma with suspected angiotropism is shown in Fig. 1A,A’. Indeed, the melanoma cells were disposed in multi-layered aggregates along the external (abluminal) surface of a dermal microvascular channel at the advancing front of the primary tumour. The surrounding dermal collagen was stained in red (hematoxylin eosin staining). Melanoma cells (black arrowheads) directly aligned along the surfaces of the endothelial cells (identified by red arrows), showed a “pericytic” location. The remainder of melanoma cells displayed a concentric multi-layered appearance. Of note, intravasation of melanoma cells was not observed.

In order to characterise the capacity of cutaneous melanoma cells to spread along the external vascular surfaces, we monitored for up to 24 hours the behaviour of cutaneous melanoma C8161-GFP cells co-cultured in Basement Membrane Extracts (BME) with HUVEC. Real time imaging of this co-culture exhibited migration of cutaneous melanoma cells towards and along endothelial cells (Fig. 1B–F and Supplementary Video S1). Cutaneous melanoma cells began to attach to the tubular network just few hours after plating, as indicated by white arrows in Fig. 1B. Twenty-four hours after plating, 81.8% of tumour cells (27 out of 33) were distributed along the tubules, while only the 18.2% of them (6 out of 33) remained dispersed in the gel (yellow arrows, Fig. 1C). Notably, we also observed unique changes in the morphology of migrating melanoma, such as the formation of protrusions and the adoption of a more tapering shape (Fig. 1D–F and Supplementary Video S1). These morphological changes were not seen in stationary melanoma cells which remained rounded.

### Angiotropism and extravascular migratory metastasis in uveal melanoma

**Figure 2:**
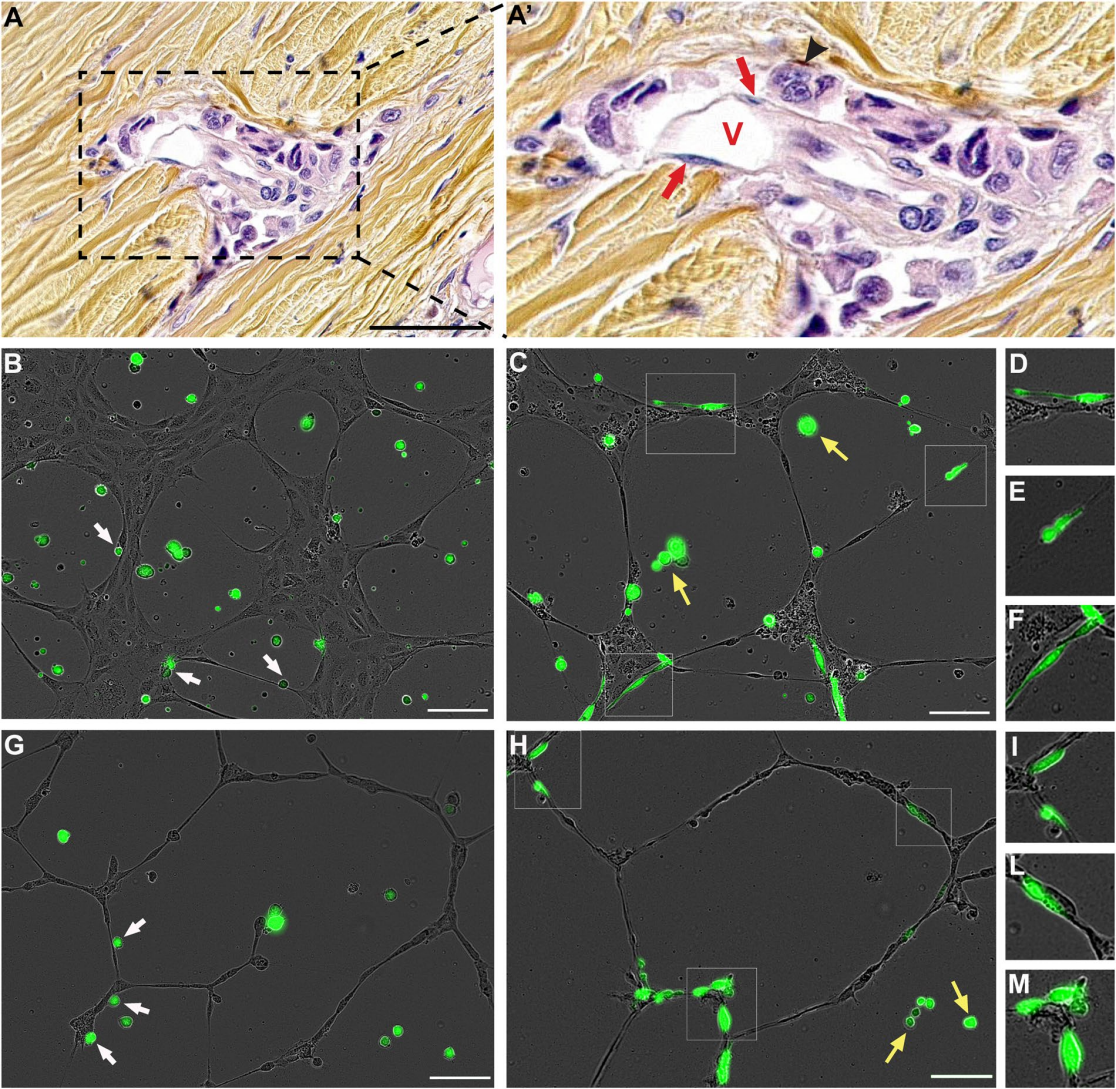
Angiotropism and extravascular migratory metastasis in uveal melanoma. (**A**) and (A’) Angiotropism in recurrent primary uveal melanoma. In this field, angiotropic melanoma cells (black arrowhead) are disposed in a concentric multi-layered pattern around the external endothelial surface of a small microvascular channel. The surrounding collagen-rich sclera of the ocular globe was stained in yellow-brown (hematoxylin eosin saffron staining). No melanoma cells are present within the vascular lumen, i.e. intravasation is absent. Scale bar is 50 µm, red V show the lumen of the vessel, black arrowheads show melanoma cells and red arrows show endothelial cells. (**B**–**F**) OMM 2.3-GFP cells co-cultured with HUVEC Endothelial cells on BME. (**G**–**N**) OMM 2.5-GFP cells co-cultured with HUVEC Endothelial cells on BME. (**B**,**G**) Picture taken 2 h after the plating of melanoma cells. (**C**,**H**) Picture taken 24 h after the plating of melanoma cells, demonstrating the spreading of both OMM 2.3-GFP and OMM 2.5-GFP cells along the endothelial tubules. (**D**–**F**) and (**I**–**M**) are -in images of angiotropic cells showed respectively in image (**C**) and (**H**), in squares. (**B**–**M**) Time-lapse images of a 24-hour video. Scale bar is 100 µm, green cells are uveal melanoma cells, grey cells are endothelial cells, white arrows show melanoma cells already attached to tubules at 2 h, yellow arrows show melanoma cells that did not attach to tubules, remaining rounded in the BME.

A clinical case of a primary uveal melanoma with suspected angiotropism is shown in Fig. 2A,A’. Microscopic examination of the sample showed angiotropic melanoma cells (black arrowheads) aligned along the external (abluminal) surface of a vascular channel within the sclera of the ocular globe. This channel was lined by a single cellular layer of endothelium, identified by red arrows. The surrounding collagen-rich sclera of the ocular globe was stained in yellow-brown (hematoxylin eosin saffron staining). The melanoma cells positioned along the surfaces of the endothelial cells showed a “pericytic” location. As in the case of cutaneous melanoma, intravasation of melanoma cells was not detected.

In order to evaluate whether uveal melanoma cells could also migrate along the external vascular surfaces, we employed the 3D co-culture approach as described above. In line with what we observed for C8161-GFP cells, real time imaging of OMM 2.3-GFP or OMM 2.5-GFP cells co-cultured in BME with HUVEC cells, exhibited migration of uveal melanoma cells towards and along endothelial cells (Fig. 2B–M and Supplementary Videos S2 and S3). Indeed, the behaviour of both uveal melanoma cell lines was very similar to the one of cutaneous melanoma. Twenty-four hours after plating, the percentage of OMM 2.3-GFP and OMM 2.5-GFP cells disposed along the tubules was 77.8% (21 out of 27) and 81.2% (26 out of 32) respectively. Melanoma cells migrating on the tubular network showed an elongated and fusiform shape, as displayed in the zoomed-in images in Fig. 2D–F and Fig. 2I–M. As in the cutaneous melanoma case, the change of morphology was not observed in uveal melanoma cells, which remained stationary in the BME (yellow arrows, Fig. 2C,H).

### Cutaneous and uveal melanomas show similar migration properties *in vitro*

**Figure 3:**
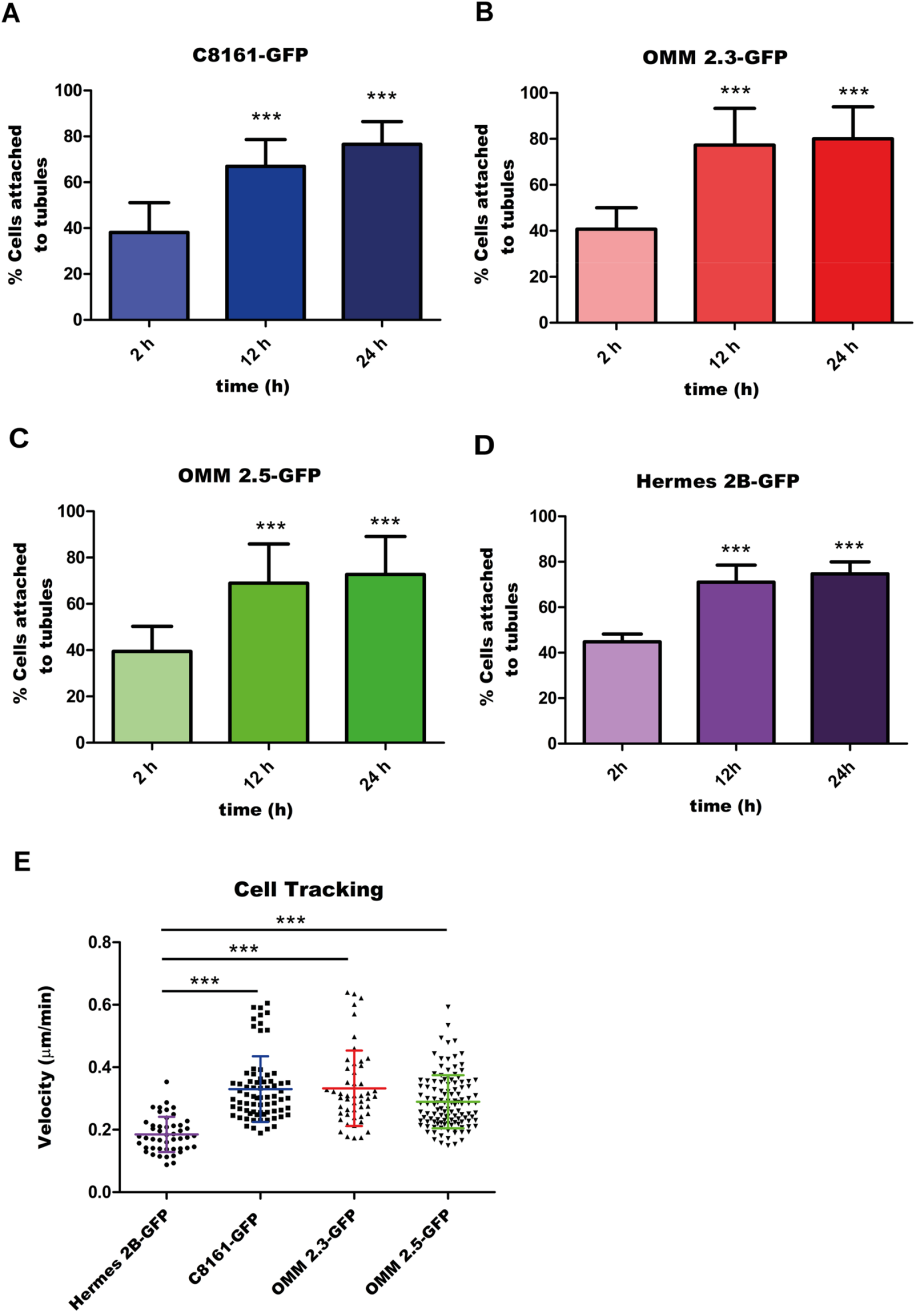
Cutaneous and uveal melanoma cells show similar migration properties *in vitro*. (**A**–**D**) Quantification of the percentage of melanoma cells attached to endothelial tubules, 2 h, 12 h and 24 h after the plating. Values shown represent the average percentage of cells attached to tubules, calculated for at least 9 videos, ± standard deviation. Statistical significance was determined using a one-way analysis of variance (ANOVA), followed by a Tukey multiple comparison test. ***P < 0.001. (**E**) Quantification of non-malignant melanocyte, cutaneous and uveal melanoma cell velocities on HUVEC tubules. The average velocity (µm/min) of each cell line is shown with coloured line as calculated for at least 48 manual tracks using the Manual Tracking plug-in of the ImageJ software. Smaller lines of the same colour display the standard deviation. Statistical significance was determined using a one-way analysis of variance (ANOVA), followed by a Tukey multiple comparison test. ***P < 0.001.

We used IncuCyte S3 Live-Cell microscope to obtain several real-time videos of the 3D co-culture of melanoma cells/non-malignant melanocytes with endothelial cells. We then performed quantitative analysis of the migration properties of cutaneous, uveal melanoma and non-malignant melanocytes. First, we evaluated the percentage of tumour cells disposed along endothelial tubules 2, 12 and 24 hours after plating (Fig. 3A–D). For this purpose, at least 9 videos for each cell line were analysed. As shown in Fig. 3A, 2 hours after plating (starting time of all the videos), 38.13% of C8161-GFP cells were attached to the tubular structures, indicating high affinity between endothelial cells and melanoma cells. The percentage of cells disposed along the tubules was 66.96% at 12 hours and reached 76.58% 24 hours after plating (n = 16). These values are highly significant (*P* < 0.001) when compared to the percentage of cells attached to tubules during the first time-lapse of the video (2 hours). As shown in Fig. 3B, 40.73% of OMM 2.3-GFP cells were attached to the endothelial tubules after 2 hour, while 77.25% and 80.07% of cells were attached at 12 and 24 hours, respectively (n = 9).

Figure 3C shows the migration properties of a second uveal melanoma cell line, OMM 2.5-GFP. For this cell line, the fraction of melanoma cells distributed along the tubules at 2 hours was 39.48% and it increased up to 68.95% and 72.74% at 12 and 24 hours after plating, respectively (n = 13). In both uveal melanoma cell lines, the values obtained at later times were highly significant if compared with the first time-lapse (*P* < 0.001).

Figure 3D shows that the fraction distributed along the tubules of the non-malignant melanocytes Hermes 2B-GFP was 44.81% at 2 hours and increased up to 71.01% and 74.73% at 12 and 24 hours after plating, respectively (n = 10). Also in this case, the values obtained at later times were highly significant if compared with the first time-lapse (*P* < 0.001).

Therefore, the results obtained for cutaneous and uveal melanoma were comparable to the ones obtained for Hermes 2B-GFP, probably due to similar angiotropic properties of these cells.

We then quantified the velocity of melanoma cells involved in extravascular migration and compared it to the velocity of non-malignant melanocytes. For this purpose, the average velocity of at least 48 cells for each cell line was calculated, employing the Manual Tracking plug-in of the ImageJ software. As shown in Fig. 3E, the average values calculated for Hermes 2B-GFP, C8161-GFP, OMM 2.3-GFP and OMM 2.5-GFP cells were respectively 0.18 ± 0.056 µm/min (n = 48), 0.33 ± 0.1 µm/min (n = 73), 0.33 ± 0.12 µm/min (n = 50) and 0.29 ± 0.85 µm/min (n = 120). The migration velocities of melanoma cell lines were similar among each other and significantly different from the one of non-malignant melanocytes.

### Angiotropism of cutaneous and uveal melanomas in zebrafish xenograft

We then wanted to compare the different migratory potential of melanoma cells and non-malignant melanocytes *in vivo*. For this purpose, C8161-GFP or Hermes 2B-GFP cells were injected in the yolk of zebrafish embryos two days post-fertilization (dpf), and the differential migration was evaluated via live imaging after 2–3 days post-injection (dpi). The experiment was performed 11 times using around 50 embryos each time, in the case of cutaneous melanoma, and 3 times injecting at least 60 embryos, in the case of non-malignant melanocytes. The strong migratory potential of uveal melanoma had already been evaluated in zebrafish embryos injected with OMM 2.3 or OMM 2.5 cells^22^. At 2/3-dpi only 50% of larvae injected with Hermes-GFP still displayed green fluorescence, while for the others no signal was detected. Additionally, migration outside the yolk cavity was not observed in larvae where green fluorescent cells were still detected. In striking contrast with what observed in non-malignant melanocyte xenotransplants, embryos injected with cutaneous melanoma showed cell migration, already after 30 hours from the injection.

**Figure 4:**
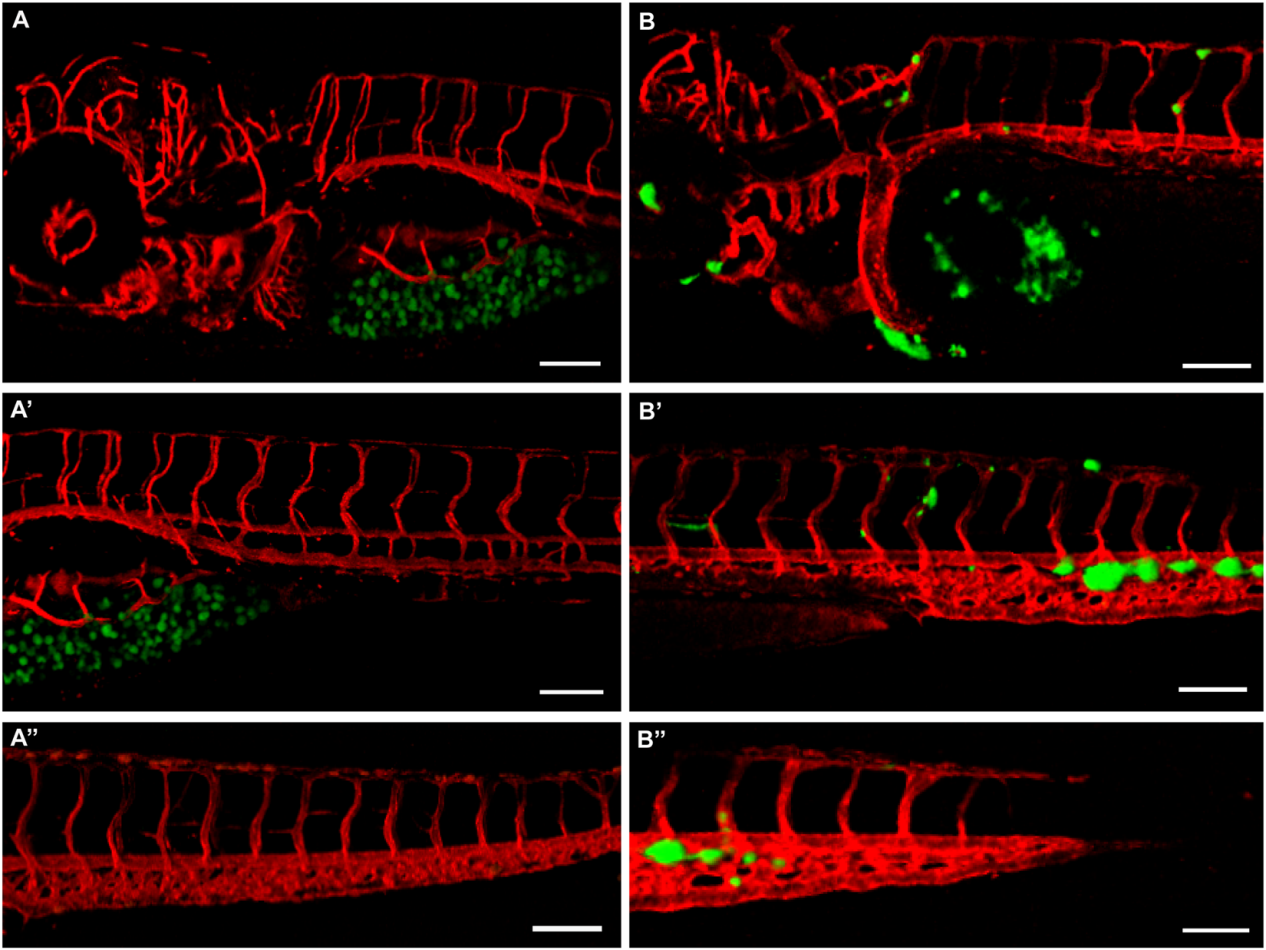
Cutaneous melanoma cells and non-malignant melanocytes show different migratory properties in zebrafish. (**A**), (A’) and (A”) Different images of a 3 dpi larva injected with Hermes-GFP cells, showing no melanocytes outside the yolk cavity. (B), (B’) and (B”) Different images of a 2 dpi embryo injected with C8161-GFP cells, showing numerous melanoma cells spread all over the body of the fish. Pictures were taken with a 10 × dry objective, employing a Zeiss LSM 700 confocal microscope. Scale bar is 50 µm, green shows melanocytes, red shows zebrafish blood vessels.

A representative example of the comparison between a 3-dpi zebrafish larva injected with Hermes 2B-GFP melanocytes and a 2-dpi one injected with C8161-GFP cells is shown in Fig. 4. As displayed by the three images on the left (Fig. 4A-A”), non-malignant melanocytes remained confined in the yolk cavity, without spreading in the rest of the fish. By contrast, the images on the right (Fig. 4B-B”), show several cutaneous melanoma cells located in different parts of the embryo, such as for the eye, the heart and the tail fin.

**Figure 5:**
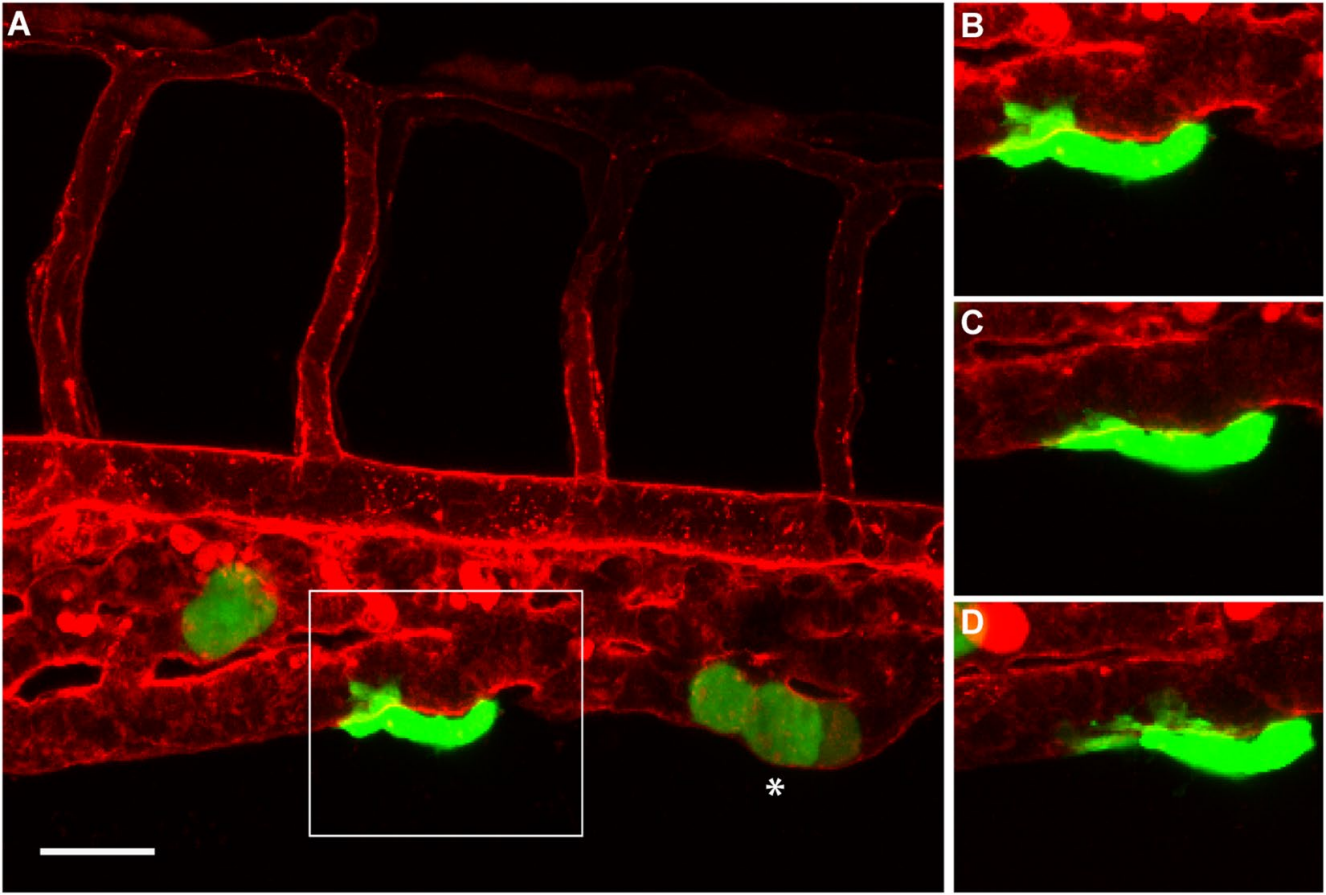
Angiotropism in zebrafish xenograft of cutaneous melanoma. (**A**) A larva injected with C8161-GFP cells, displaying an angiotropic cell (in the square) extending along the external surface of the caudal vein. (**B**–**D**) are time-lapse images of the same angiotropic cell taken at 0, 4 and 8 hours after the beginning of the imaging. The images were obtained employing a Zeiss LSM 700 confocal microscope (25 × oil objective), starting from 30 hours post injection. Scale bar is 20 µm, green shows cutaneous melanoma cells, white asterisk shows intravascular melanoma cells, red shows zebrafish blood vessels.

We then wondered whether we could detect angiotropism and pericytic mimicry in embryos displaying C8161-GFP cells outside of the yolk sac. Therefore, the interactions between cutaneous melanoma cells and the external surface of zebrafish vessels were monitored via live imaging coupled with 3D reconstruction, starting from 30 hours after the injection. Figure 5 shows a representative example of a zebrafish larva displaying a striking angiotropic cell (indicated by the square). Indeed, this tumour cell was wrapped around the caudal vein of the larva, attached to the abluminal surface of the vessel. Interestingly, this cell showed unique changes in the morphology, such as the formation of pseudopodial protrusions and the adoption of a tapering shape. As displayed by the three time points presented (0, 4 h and 8 h) in Fig. 5B–D and the Videos S5 and S6, this cell exhibited cellular processes extending along the external surface of the caudal vein of the larva. During the 10-hour video, the angiotropic cell was slowly gliding on the surface of the vessel, without entering into the circulation. The 3D reconstruction of this video (Supplementary Video S7) confirmed the existence of a slow crawling movement of the melanoma cell on the vessel surface, implying pericytic mimicry and EVMM. Other examples of angiotropic cutaneous melanoma cells surrounding the zebrafish vessels are shown in Supplementary Fig. S4.

Intravascular tumour cell migration was also detected (Fig. 5A, white asterisk). This group of cells seems to adhere to the internal surface of the vessels, slowly crawling on it. Of note, the time-lapse frames showed in Supplementary Video S5 do not allow detecting tumour cells circulating within the blood flow.

**Figure 6:**
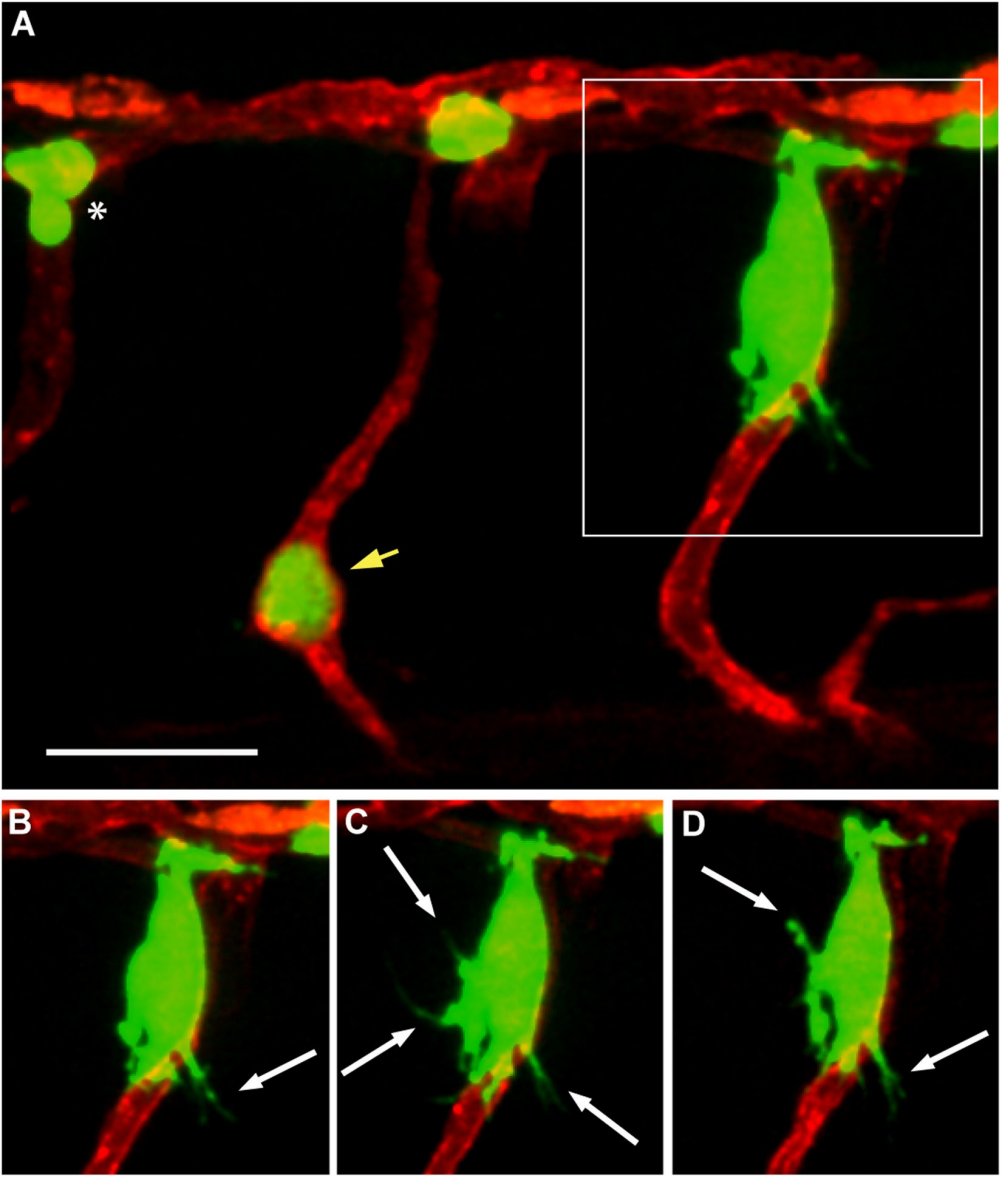
Angiotropism in zebrafish xenograft of uveal melanoma. (**A**) A larva injected with OMM 2.3-GFP cells, displaying a micrometastasis of angiotropic cells (in the square) cuffing the external surface of an intersegmental vessel. (**B**–**D**) are time-lapse images of the same angiotropic cells taken at time 0, 4 and 8 hours after the beginning of the imaging. The images were obtained employing a Zeiss LSM 880 confocal microscope (40 × water objective), starting from 30 hours post injection. Scale bar is 20 µm, green shows melanoma cells, red shows zebrafish blood vessels, white arrows show pseudopodial protrusions formed by angiotropic cells, white asterisk shows intravascular melanoma cells, yellow arrow shows melanoma cells trapped in an intersegmental vessel.

We then investigated whether angiotropism could also be detected in 2-dpf zebrafish embryos injected with uveal melanoma cells. As in the case of cutaneous melanoma, interactions between uveal melanoma cells and the abluminal surface of zebrafish vessels were studied employing live imaging coupled with 3D reconstruction. The experiment was performed 5 times using around 50 embryos each time. A representative example of a larva displaying a micrometastasis formed by angiotropic uveal melanoma cells is shown in Fig. 6A (indicated by the square). Interestingly, this group of cells was located outside an intersegmental vessel of the injected embryo, adhering to the external surface of the vessel (Fig. 6B–D).

In line with what we observed for cutaneous melanoma cells, the uveal melanoma angiotropic cells exhibited lengthy and fine cellular protrusions extending on the abluminal surface of the vessel and in the surrounding microenvironment (indicated by white arrows in Fig. 6B–D). As displayed in these images and in Videos S8 and S9, the micrometastasis remained located outside the intersegmental vessel without entering in the circulation.

Putative intravascular uveal melanoma cells were also detected (Fig. 6A, white asterisk). Of note, the cell indicated by the yellow arrow remained trapped inside an intersegmental vessel of the embryo, promoting the sprouting of new small horizontal vessels (Supplementary Video S8). Also in this case, the time-lapse frames showed in the video do not allow the detection of tumour cells circulating within the blood flow.

## Discussion

Malignant cells use a variety of motility and invasion mechanisms, specifically hematogenous spread, angiotropism and extravascular migratory metastasis (EVMM). It is known that aberrant expression of embryonic epithelial-mesenchymal transition (EMT) factors triggers extensive plasticity of cancer cells, including melanoma cells^31^ via EMT plasticity, invading melanoma cells can use a broad spectrum of invasion strategies depending upon many environmental determinants^32,33^ leading to tumour resistance and metastasis. Despite a large number of studies analysing various factors responsible for triggering cancer cell progression^34,35^ the genetic and epigenetic basis for invasive cancer cell strategies remains poorly understood.

In the present study, we described the presence of angiotropism and EVMM in cutaneous and uveal melanoma spread both *in vitro* and *in vivo*. Indeed, not only we evaluated and characterised angiotropism and EVMM using our already established 3D co-culture approach, but we also studied these processes *in vivo* in zebrafish larvae, via live imaging. To our knowledge, this study is the first attempt to characterise this novel mode of metastatic dissemination in such a model.

Angiotropism and pericytic mimicry have been previously demonstrated in human biopsies of common malignancies of the skin^36^, pancreas^37^ and prostate^38^. Similar findings have been shown in other solid tumours, notably in the malignant brain tumour, glioblastoma multiform. In particular, invasive glioblastoma cells are known to follow peculiar anatomic structures in the central nervous system, including the abluminal surface of blood vessels, displaying the same phenotypic pericytic mimicry as angiotropic melanoma cells. For example, Cheng *et al*. suggested a role for glioma stem cells as pericyte progenitors and proposed that they might contribute to the formation of cancer vessels and to the progression of tumour growth^39^.

In the present study, we additionally documented the similarities in the migratory properties of cutaneous and uveal melanoma cells, despite their different genetic profiles. Indeed, they both showed angiotropism and pericytic mimicry *in vivo* and *in vitro*.

Curiously, we also reported that non-malignant immortalised human melanocytes Hermes 2B-GFP, showed a similar behaviour *in vitro*. Indeed, these cells attached to the tubules formed by HUVEC cells, cultured in basement membrane extracts, displaying angiotropism.

Nevertheless, despite their angiotropic properties *in vitro*, Hermes 2B-GFP cells displayed a significantly slower migration velocity along the endothelial tubules probably linked to the non-malignancy of these cells. The average velocity of cutaneous and uveal melanoma cells moving along the vascular tubules in 3D co-culture was comparable, around 0.3 µm/min. This value is within the range of both tumour cell and neural crest cell (NCC) migration average velocities, as previously reported^7^. The analogy of EVMM with the migration of NCC in the developing embryo has already been emphasized^6^. The embryonic pathways of highly migratory NCC and their regulation during development result in the establishment of melanocytes among other cell types^40^. Cancer cells with stem/embryonic properties may contribute to the formation of tumour vasculature, consistent with the tissue organization seen in angiotropism (or vascular co-option)^3^.

The angiotropic behaviour of Hermes 2B-GFP could be explained by the mesenchymal, neural crest origin of melanocytes, as reported also in another study of our group^41^. In this previous work, melanocytes from benign nevi were able to attach and spread along capillary-like structures *in vitro*, while they could not grow *in vivo* on the chick chorioallantoic membrane. In line with these findings, we showed that no Hermes 2B-GFP cells were detected 2–3 days after injection in around 50% of zebrafish larvae. In the remaining injected larvae, the transplanted cells showed no migration and stayed confined in the yolk sac.

Hence, despite angiotropic behaviour *in vitro*, Hermes 2B-GFP cells do not show any progressive growth *in vivo*. Indeed, malignant transformation is needed for cells to migrate and invade in response to challenging *in vivo* environmental conditions.

Concerning the mode of tumour migration, we observed cutaneous and uveal melanoma cells migrating individually as single cells and as small groups of cells, as we previously showed in the murine brain melanoma model^3^. These observations could correspond to what is described as single-cell and collective migrations^32,42^.

Consistently with what we had reported in our previous study^43^ and in analogy with cancer and NCC migration, here we observed unique changes in the morphology of angiotropic melanoma cells, both *in vitro* and *in vivo*. These alterations included the formation of protrusions, the adoption of a more tapering shape and the adhesion to endothelial tracks.

There is a wide range of pseudopodia that are needed to extend the leading edge of cells during migration on both 2D surfaces and in 3D extracellular matrix. The type of protrusion can be used to describe and define a peculiar mode of cell motility. In our case, we speculated that the finger-like protrusions characterising angiotropic melanoma cells observed in 3D co-culture and in zebrafish xenografts, could be filopodia. Indeed, filopodia are described as actin-rich finger-like pseudopodia at the leading edge of cells, which are able to sense the local microenvironment. They can operate alone or combined with other protrusions such as for example lobopodia or lamellipodia in 2D and 3D environments^44^. While in line with these reported results, our observations are only based on the morphology of the pseudopodia observed in the videos recorded and a more detailed characterisation of these protrusions is thus required.

In conclusion, here we provide evidence of angiotropism in two types of melanoma cells, for the first time in an *in vivo* model. This behaviour could contribute to the metastatic dissemination via a non-canonical pathway (EVMM), in addition to the classical intravascular pathway of cancer cells migration. These two mechanisms could act *in vivo* as non-mutually exclusive, but rather synergistic ways of tumour dissemination.

## Electronic supplementary material

Combined Supplemetary Info

Supplementary Video 1

Supplementary Video 2

Supplementary Video 3

Supplementary Video 5

Supplementary Video 6

Supplementary Video 7

Supplementary Video 8

Supplementary Video 9
