# Supplementary material for: Angiotropism and extravascular migratory metastasis in cutaneous and uveal melanoma progression in a zebrafish model
Source: Sci Rep. 2018 Jul 11;8:10448. doi: 10.1038/s41598-018-28515-6 (PMC6041265; doi:10.1038/s41598-018-28515-6)
Supplement: Supplementary file 1 — Combined Supplemetary Info [file 41598_2018_28515_MOESM1_ESM.pdf]

Angiotropism and Extravascular Migratory Metastasis in Cutaneous and Uveal Melanoma Progression in a Zebrafish Model

Giulia Fornabaio [1,2,3], Raymond L. Barnhill [4,5], Claire Lugassy [2], Laurent A. Bentolila [6,7], Nathalie Cassoux[5,8], Sergio Roman-Roman [2], Samar Alsafadi[2,*], Filippo Del Bene[1,2,*]

* Correspondence to: samar.alsafadi@curie.fr, filippo.del-bene@curie.fr

[1]Institut Curie, PSL Research University, Inserm U934, CNRS UMR315 F-75005, Paris, France
[2]Institut Curie, PSL Research University, Department of Translational Research, F-75005, Paris, France
[3]Sorbonne Universités, UPMC Univeristy Paris 6, CNRS UMR 3215, INSERM U934, F-75005, Paris, France
[4] Institut Curie, PSL Research University, Department of Pathology, F-75005, Paris, France
[5] Faculty of Medicine, University of Paris Réné Descartes, F-75006 Paris, France
[6] California NanoSystems Institute, Los Angeles, CA, 90095, USA
[7] Department of Chemistry and Biochemistry, University of California, Los Angeles, CA, 90095, USA
[8] Institut Curie, PSL Research University, Department of Opthalmology, F-75005, Paris, France.

Supplementary material legends

Supplementary Video S1. Video showing EVMM of C8161-GFP cutaneous melanoma cells co-cultured with HUVEC endothelial cells (24 hour video, 20x dry objective, acquisition time: 15 minutes).

Supplementary Video S2. Video showing EVMM of OMM 2.3-GFP uveal melanoma cells co-cultured with HUVEC endothelial cells (24 hour video, 20x dry objective, acquisition time: 15 minutes).

Supplementary Video S3. Video showing EVMM of OMM 2.5-GFP uveal melanoma cells co-cultured with HUVEC endothelial cells (24 hour video, 20x dry objective, acquisition time: 15 minutes).

Supplementary Image S4. Other examples of angiotropic cutaneous melanoma cells cuffing the vessels of zebrafish embryos. (**A**) A 30 hpi embryo, imaged with a 25x oil objective, using a Zeiss LSM 700 confocal microscope. (**B**) A 30 hpi embryo, imaged with a 40x water objective, using a Zeiss LSM 700 confocal microscope. (**C**) and (**D**) 4 dpi embryos, imaged with a 63x water objective, using a Zeiss LSM 880 confocal microscope. (**A**), (**B**), (**C**) and (**D**) white bar= 20 μm, white arrows= angiotropic cells, green= melanoma cells, red= zebrafish blood vessels.

Supplementary Video S5. Video showing an angiotropic cutaneous melanoma cell cuffing a vessel (25x oil objective, 25 minute acquisition, 10 hour video, 30 hpi embryo).

Supplementary Video S6. A zoomed-in video of angiotropic cell of Supplement S5.

Supplementary Video S7. 3D reconstruction of the video in Supplement S6 (obtained using the software Bitplane-Imaris).

Supplementary Video S8. Video showing angiotropic uveal melanoma cells attached to the external surface of vessels (40x water objective, 20 minute acquisition, 8 hour video, 30 hpi embryo).

Supplementary Video S9. A zoomed-in video of angiotropic cells of Supplement S8.

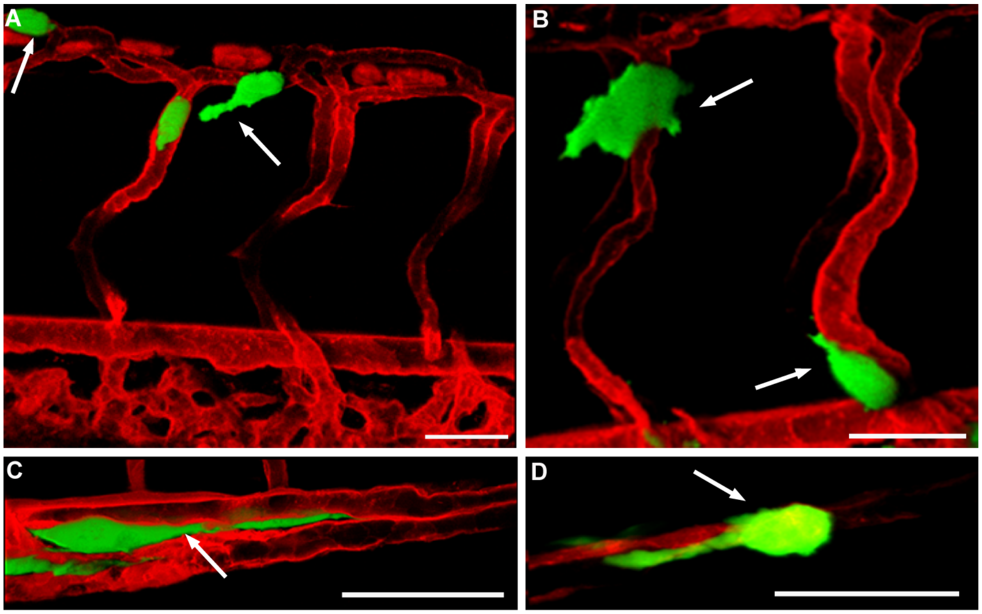

Supplementary figure S4
